# UAV NDVI-Based Vigor Zoning Predicts PR-Protein Accumulation and Protein Instability in Chardonnay and Sauvignon Blanc Wines

**DOI:** 10.3390/plants15020243

**Published:** 2026-01-13

**Authors:** Adrián Vera-Esmeraldas, Mauricio Galleguillos, Mariela Labbé, Alejandro Cáceres-Mella, Francisco Rojo, Fernando Salazar

**Affiliations:** 1 Industrial Fermentation Laboratory, Escuela de Alimentos, Facultad de Ciencias Agronómicas y de los Alimentos, Pontificia Universidad Católica de Valparaíso, Av. Waddington 716, Valparaíso 2340000, Chile; 2 Facultad de Ingeniería y Ciencias, Universidad Adolfo Ibáñez, Diagonal Las Torres, 2640, Peñalolén, Santiago 7941169, Chile; 3 Escuela de Agronomía, Facultad de Ciencias Agronómicas y de los Alimentos, Pontificia Universidad Católica de Valparaíso, Valparaíso 2340053, Chile; 4 Bioeconomy Science Institute Ltd., Cropping Systems and Environment, Hawke’s Bay, Lincoln 7608, New Zealand

**Keywords:** precision viticulture, unmanned aerial vehicles (UAV), NDVI, pathogenesis-related proteins, protein haze, bentonite

## Abstract

Protein instability in white wines is mainly caused by pathogenesis-related (PR) proteins that survive winemaking and can form haze in bottle. Because PR-protein synthesis is modulated by vine stress, this study evaluated whether unmanned aerial vehicle (UAV) multispectral imagery and NDVI-based vigor zoning can be used as early predictors of protein instability in commercial Chardonnay and Sauvignon Blanc wines. High-resolution multispectral images were acquired over two seasons (2023–2024) in two vineyards, and three vigor zones (high, medium, low) were delineated from the NDVI at the individual vine scale. A total of 180 georeferenced vines were sampled, and musts were analyzed for thaumatin-like proteins and chitinases via RP-HPLC. Separate microvinifications were carried out for each vigor zone and cultivar, and the resulting wines were evaluated for protein instability (heat test) and bentonite requirements. Low-vigor vines consistently produced musts with higher PR-protein concentrations, greater turbidity after heating, and higher bentonite demand than high-vigor vines, with stronger effects in Sauvignon Blanc. These vigor-dependent patterns were stable across vintages, despite contrasting seasonal conditions. Linear discriminant analysis using NDVI, PR-protein content, turbidity, and bentonite dosage correctly separated vigor classes. Overall, UAV NDVI–based vigor zoning provided a robust, non-destructive tool for identifying vineyard zones with increased risk of protein instability. This approach supports precision enology by enabling site-specific stabilization strategies that reduce overtreatment with bentonite and preserve white wine quality.

## 1. Introduction

In white winemaking, one of the primary enological challenges is protein instability, which manifests as haze formation during storage or after bottling. This phenomenon is primarily caused by the presence of pathogenesis-related (PR) proteins, particularly thaumatin-like proteins (TLPs) and chitinases (Chit) [1]. These PR proteins are the principal contributors to haze, as they remain stable throughout fermentation due to their resistance to heat and proteolysis [2]. Even minimal haze formation is considered a major sensory and commercial defect, forcing winemakers to routinely evaluate protein stability and apply clarification treatments before bottling [3].

The PR proteins responsible for haze formation, mainly TLPs and chitinases, originate in the grape berry and are synthesized as part of the plant’s defense system [1,4]. Their accumulation is strongly modulated by stress-related regulation during ripening, particularly through changes in canopy microclimate such as temperature, light exposure, and humidity, all of which are closely linked to vine vigor. Variations in canopy density alter these microclimatic conditions and may therefore influence the extent to which berries activate stress-induced PR-protein pathways. Although this relationship has been suggested conceptually, no study has quantified how spatial variability in vigor translates into differences in PR-protein accumulation at the vineyard scale [5,6]. These proteins remain exceptionally stable under winemaking conditions resistant to enzymatic degradation and soluble at wine pH, allowing them to persist into the bottled product and generate haze even after fermentation [5]. Importantly, PR-protein concentrations vary markedly across cultivars, seasons, and microclimatic environments [6]. For example, Sauvignon Blanc typically exhibits higher TLP levels and greater susceptibility to haze development than other white varieties. Understanding these cultivar-specific differences in susceptibility to PR-protein accumulation is essential for identifying vineyard factors that contribute to final wine instability.

Given the critical role of PR proteins in haze formation and their pronounced variability, winemakers routinely implement clarification strategies to reduce their concentration before bottling. Among these, bentonite fining is the most widely employed technique in commercial white winemaking. Bentonites are negatively charged clay minerals that bind positively charged wine proteins, including thaumatin-like proteins and chitinases, promoting their precipitation and removal [7]. Despite its efficacy and relative affordability, this approach presents important drawbacks. Bentonite can also remove aromatic and flavor-active compounds essential for wine sensory expression [8], while its use generates substantial lees that reduce wine volume and increase processing costs [7,9].

A major challenge arises when wines contain high initial concentrations of PR proteins, as occurs frequently in cultivars such as Sauvignon Blanc. In these cases, bentonite demand becomes disproportionately high, leading to larger additions that exacerbate wine losses, elevate operational costs, and increase the risk of stripping desirable aromas and texture. Determining the optimal dosage can also be uncertain, requiring multiple fining trials that add further time and cost to production [10]. These limitations have intensified efforts to develop more selective, efficient, and sustainable alternatives for protein stabilization.

Several alternative strategies have been developed to reduce bentonite use and preserve wine quality. Proteolytic enzymes, such as fungal-derived aspergill pepsin and bacterial BcAP8, have shown the ability to degrade TLPs and chitinases when activated correctly in controlled thermal conditions [11,12]. Other approaches include the addition of yeast-derived mannoproteins, which function as protein stabilizers by preventing haze formation without removing desirable aromatic compounds [11]. Physical separation technologies such as ultrafiltration have also demonstrated some effectiveness in selectively reducing protein content [10,13]. Despite their potential, these alternatives often face challenges related to cost, implementation, or limited efficacy and have yet to replace bentonite in commercial winemaking settings fully.

However, all of these post-fermentative treatments share a reactive rather than preventive nature. They can only be applied after instability has been detected, which limits their efficacy. Thus, the current challenge lies in developing early, vineyard-level tools capable of predicting which areas of a block are more prone to producing unstable wines. By anticipating instability risk before harvest, winemakers could optimize stabilization strategies, reducing excessive fining to preserve wine integrity. In other words, the key lies in predicting early which vineyard zones are likely to present a higher risk of protein instability based on physiological and environmental indicators of stress.

This context underscores the critical need for vineyard-level tools capable of anticipating protein instability risks before harvest. Traditionally, winemakers rely on laboratory heat tests performed on musts or finished wines to determine protein instability and adjust bentonite dosage. However, these analyses can only be conducted after harvest, when PR-protein concentrations are already fixed, which limits the efficacy of stabilization decisions and often forces wineries to react under time pressure during early vinification. In contrast, because PR-protein accumulation is closely linked to vine physiological stress, water availability, and canopy microclimate, UAV-based spectral imaging offers a non-invasive way to detect these stress patterns in situ and prior to harvest. Mapping spatial variability in vigor using vegetation indices such as NDVI or NDRE provides early insight into which vineyard zones are more likely to accumulate higher levels of haze-forming proteins. This information enables winemakers to plan stabilization operations proactively, for example, by allocating larger bentonite doses to high-risk zones or preparing differential fining strategies, thereby improving process efficacy while reducing unnecessary fining in low-risk areas. Furthermore, knowledge of vigor-dependent risk can support harvest logistics by allowing growers to adjust picking schedules for zones with expected peak PR-protein levels. The integration of remote sensing with enological decision-making represents a practical advancement in precision viticulture, where spatial vineyard information can directly inform cellar strategies to enhance wine stability and overall production sustainability.

Spatial differences in vine vigor driven by soil heterogeneity, water availability, and canopy microclimate can strongly influence berry metabolism and, consequently, grape and wine composition. Previous research has shown that vine vigor correlates with variations in berry size, phenolic content, nitrogen composition, and aromatic potential [14,15]. For instance, Ferrer et al. [16] demonstrated that NDVI-based vigor maps were related to yield and grape composition in humid climates; Matese et al. [17] associated UAV-derived vigor zones with anthocyanin content in grapes; and Dorin et al. [18] reported that vigor heterogeneity influenced must quality and fermentation performance in Riesling. Despite these advances, no study has directly evaluated the relationship between vigor and PR-protein accumulation and its enological potential, leaving a significant gap in understanding how vineyard variability translates into protein instability risk.

In recent years, the maturation of precision agriculture technologies has opened new opportunities to address vineyard variability in ways that were previously impractical. Remote sensing systems based on unmanned aerial vehicles (UAVs) equipped with multispectral sensors now provide high-resolution, non-destructive monitoring of vine physiological status, enabling growers to capture spatial differences that are often invisible through conventional scouting [19,20]. These platforms deliver spatially explicit information on key vineyard attributes, including canopy vigor, water status, leaf area index (LAI), above-ground biomass, and phenological development, and they can be updated throughout the season [21,22]. The ability to compute vegetation indices such as NDVI, GNDVI, and NDRE from UAV imagery further strengthens this approach, as these indices serve as reliable proxies for photosynthetic activity and canopy structure. As a result, UAV-based multispectral imaging has become a robust and accessible tool for delineating vigor-based zones within vineyard blocks and for linking physiological heterogeneity with agronomic or enological outcomes [23].

Although several vegetation indices are available for characterizing vine vigor, their sensitivity varies depending on canopy structure and leaf area index. NDRE, which exploits the red-edge region, has been reported to maintain sensitivity under very dense canopies where NDVI may approach saturation, particularly in vigorous vineyards [24]. However, NDVI remains the most widely adopted index in viticulture due to its robustness, extensive validation across cultivars and environments, and strong physiological interpretability [25]. Importantly, NDVI integrates canopy density, photosynthetic activity, and water status, all of which are closely linked to vine stress responses and yield variability. While NDVI saturation can occur under extremely high leaf area index conditions, this limitation is less critical in vertically trained vineyards such as VSP systems, where canopy density remains within a functional dynamic range [15,24]. In the present study, preliminary analyses confirmed that NDVI consistently showed stronger associations with grape maturity (°Brix) than NDRE across cultivars and seasons, supporting its selection as the primary index for vigor zoning with direct enological relevance.

Numerous studies have demonstrated the effectiveness of UAV-derived spectral indices in detecting spatial variability within vineyard plots, facilitating site-specific management practices based on physiological indicators rather than on visual assessments or topographic features [26,27]. These indices have been significantly correlated with agronomic and enological variables such as total soluble solids (°Brix) [14], titratable acidity, pH, anthocyanin concentration [17], berry size, and yield. Moreover, vegetation indices such as NDVI and NDRE have also been applied to assess plant health status and estimate the risk of disease development in dense canopies [28], where reduced airflow can favor fungal infections such as Botrytis cinerea [29]. The integration of UAV-based multispectral imagery with photogrammetric and spectral analysis tools has broadened the applicability of this technology in viticultural decision-making. Recent investigations have demonstrated that vigor maps generated from multi-temporal UAV data are not only helpful in monitoring vegetative growth but also for predicting spatial patterns related to grape composition and wine potential.

Compared to conventional field scouting or satellite-based monitoring [30], UAV-based multispectral imaging combines high spatial detail, flexible deployment, and cost-effectiveness, making it particularly suitable for frequent assessments during the growing season. This capability enables viticulturists to detect subtle physiological variations across the vineyard and implement targeted interventions aligned with specific phenological stages. The resulting vigor maps enable the delineation of optimal management zones to support variable-rate applications focused on irrigation, fertilization, or selective harvesting [31]. Beyond agronomic uses, these high-resolution datasets also provide valuable inputs for anticipating enological traits, including ripeness progression, phenolic development, and protein stability, enhancing the precision of vineyard management and final wine quality [32].

In this study, NDVI was selected as the principal spectral indicator due to its demonstrated sensitivity to water and nitrogen stresses, two physiological factors closely linked to the upregulation of PR-protein synthesis through stress-related pathways such as abscisic acid (ABA), ethylene, and salicylic acid signaling. NDVI remains one of the most widely adopted vegetation indices in viticulture because of its robustness under variable illumination, reduced soil background sensitivity at dense canopy cover, and extensive validation across vineyard systems [28,32]. While other indices such as NDRE and GNDVI can also capture canopy vigor, NDVI provides a consistent and interpretable metric that facilitates temporal and cross-site comparisons. Preliminary analyses in this work further confirmed that NDVI exhibited the strongest correlation with grape maturity (°Brix) among the indices tested, reinforcing its suitability for vigor-based zoning with enological relevance.

Therefore, this study addresses the existing knowledge gap regarding how spatial variability in vine vigor relates to PR-protein accumulation and subsequent wine protein instability. We hypothesized that spatial differences in vine vigor, detected through NDVI-based UAV multispectral imaging, reflect underlying physiological stress patterns that modulate PR-protein synthesis in grape berries and ultimately influence protein instability risk in white wines.

Accordingly, the main objective of this research was to quantitatively link NDVI-based vigor zoning with PR-protein concentrations in grape juice and to evaluate how these spatial differences translate into wine protein instability and bentonite fining requirements. By integrating UAV-derived vigor maps with analytical PR-protein quantification and micro-vinification assays, this work proposes a spatially explicit framework connecting vineyard vigor heterogeneity with enological protein stability. This approach provides a foundation for precision viticulture strategies aimed at optimizing protein stabilization practices, reducing excessive bentonite use, and improving overall wine quality.

## 2. Results and Discussion

### 2.1. Spatial Variability of Vine Vigor Across Seasons and Cultivars

**Figure 1:**
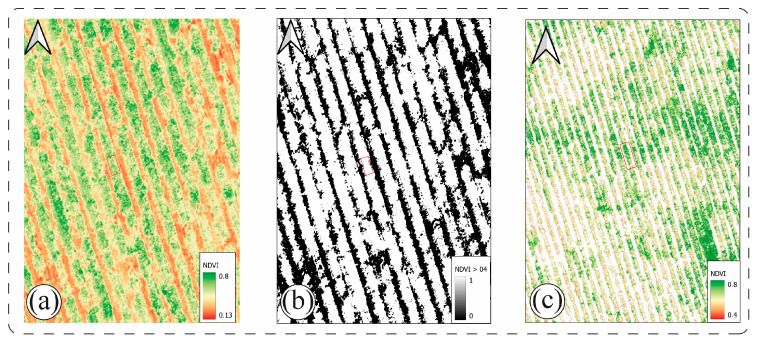
Workflow for vine-level NDVI segmentation and extraction using UAV multispectral imagery. (**a**) NDVI map derived from the red and near-infrared (NIR) bands, with values ranging from 0.13 to 0.80, highlighting spatial variability in canopy vigor. (**b**) Binary vegetation mask created using an NDVI threshold of >0.4 to separate actively photosynthetic vegetation (black) from non-vegetative areas (white). (**c**) Spatial sampling grid (1.20 × 1.00 m) overlaid on the filtered NDVI image, with plant-level polygons (red squares) used to extract mean NDVI values per vine. The red squares delineate individual vine canopy areas within the grid framework. This step enabled direct linkage between spectral vigor and individual plant traits.

The segmentation workflow used to extract vine-level NDVI values is illustrated in Figure 1 and constitutes a critical step in accurately representing the spatial distribution of canopy vigor. By integrating structural information from the canopy height model (CHM) with spectral thresholds derived from NDVI, the method effectively minimized interference from soil, trellis wires, and interrow shadows, an approach consistent with previous segmentation strategies employed in UAV-based viticulture. In Chardonnay, a CHM threshold of 0.50 m combined with NDVI > 0.40 proved robust for both seasons. In contrast, Sauvignon Blanc required adaptive thresholds due to its inherently more open canopy architecture and lower leaf area index, a characteristic highlighted for this cultivar under water-limited conditions [22]. In 2024, reduced vegetative development required lowering the thresholds to 0.40 m for canopy height and NDVI > 0.35 to avoid underestimation of true canopy area and ensure accurate extraction of plant-level spectral information. These cultivar-specific adjustments underline the importance of tailoring segmentation criteria to canopy structure, an aspect rarely addressed explicitly in previous UAV studies.

**Figure 2:**
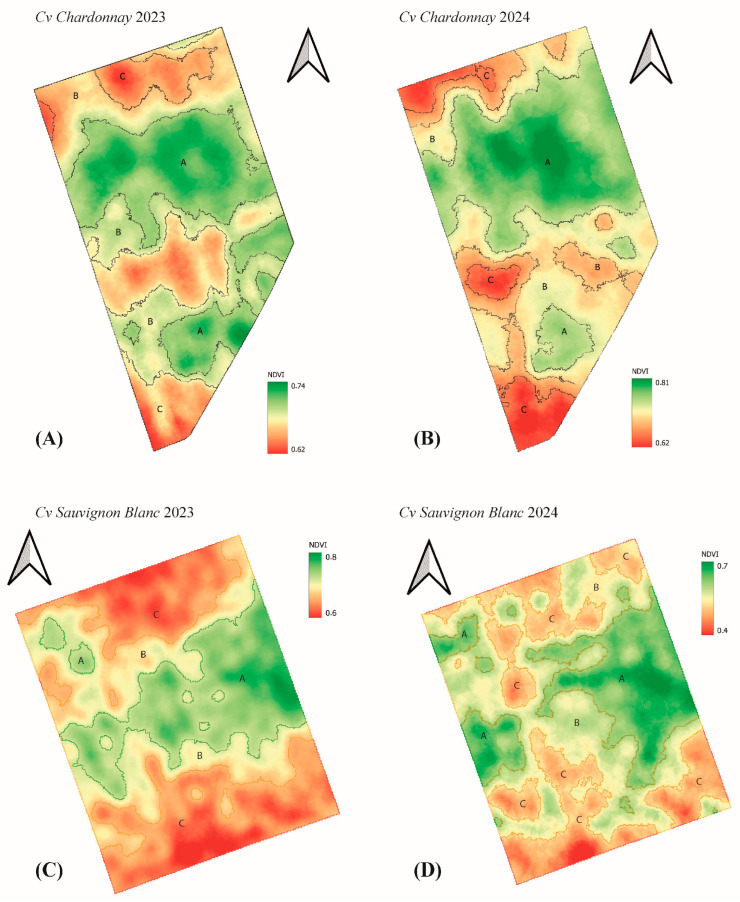
Spatial distribution of vine vigor based on NDVI across cultivars and growing seasons. Normalized Difference Vegetation Index (NDVI) maps derived from UAV-based multispectral imagery illustrate the spatial heterogeneity of vine vigor in two white grape cultivars Chardonnay and Sauvignon Blanc across the 2023 and 2024 growing seasons. Panels (**A**) and (**B**) correspond to Chardonnay, while panels (**C**) and (**D**) represent Sauvignon Blanc. For each cultivar, high-vigor zones (Zone A) are shown in green tones, medium-vigor zones (Zone B) in yellow, and low-vigor zones (Zone C) in red.

The NDVI maps (Figure 2) revealed clear and spatially structured heterogeneity across vineyard blocks, with distinct vigor gradients that evolved between the 2023 and 2024 seasons. In Chardonnay, vigor patterns remained comparatively stable over time, characterized by compact high-vigor clusters and peripheral low-vigor zones. This spatial configuration aligns with studies indicating that Chardonnay tends to maintain more consistent vegetative expression across seasons due to its denser canopy and greater tolerance to moderate hydric fluctuations [17]. The expansion of high-vigor areas in 2024 may reflect improved canopy development associated with enhanced winter rainfall, consistent with reports showing that NDVI increases with improved soil moisture availability [16,33]. These patterns suggest that Chardonnay exhibits moderate interannual variability and that NDVI serves as a reliable indicator of vegetative development.

Sauvignon Blanc, in contrast, displayed pronounced interannual changes, with an expansion of low-vigor sectors in 2023 followed by a more balanced distribution in 2024. The sharper decline in NDVI values observed in 2024 for this cultivar suggests a heightened sensitivity to localized water deficits or microclimatic fluctuations. This interpretation agrees with studies demonstrating that Sauvignon Blanc shows stronger spectral and physiological responses to hydric stress due to its thinner canopy and potentially shallower root system [34,35]. Similar variability has been described by Pádua et al. [36], who reported strong vintage-dependent spectral fluctuations in aromatic cultivars. The present results reinforce these observations, indicating that Sauvignon Blanc serves as a more responsive bioindicator of environmental stress at the vineyard scale.

The vigor classes shown in Figure 2 were generated using k-means clustering applied to vine-level NDVI data, performed independently for each cultivar and growing season. This unsupervised, data-driven approach is widely used in UAV-based vigor zoning studies [23,37], and allows the delineation of low-, medium-, and high-vigor classes by minimizing within-cluster variance while accounting for cultivar- and year-specific variations in vegetative development.

As expected in clustering-based approaches, the resulting vigor classes exhibited unequal sample sizes and spatial extents. This asymmetry reflects genuine physiological heterogeneity within the vineyard rather than a methodological limitation. High-vigor vines tended to form spatially compact clusters associated with favorable soil depth or irrigation patterns, whereas low-vigor vines were more spatially dispersed. This spatial organization is consistent with patterns previously described in high-resolution UAV-based vigor studies conducted in Mediterranean vineyard systems [38].

Overall, the integration of structural segmentation shown in Figure 1 with NDVI-based vigor mapping in Figure 2 provides a coherent and physiologically meaningful representation of canopy variability across cultivars and seasons. The spatial patterns observed are consistent with established literature showing that NDVI reliably reflects differences in soil plant atmosphere interactions, including water availability, canopy density, and microclimate effects [15]. The more dynamic vigor responses observed in Sauvignon Blanc highlight its greater susceptibility to environmental fluctuations, an aspect that becomes essential for interpreting the biochemical and enological differences described in Section 2.2 and Section 2.3. These findings confirm that UAV-derived NDVI mapping is a robust and sensitive tool for capturing cultivar-specific vigor dynamics and offers a spatial foundation for linking vegetative status with grape composition, PR-protein accumulation, and wine stability.

### 2.2. Relationship Between NDVI and Grape Maturity at Harvest

The relationship between vine vigor and grape maturity showed a consistent negative association across both cultivars and seasons. In Chardonnay, NDVI exhibited a strong and highly significant negative correlation with °Brix in 2023 (r = −0.86; R^2^ = 0.74; *p* < 0.001), indicating that low-vigor vines accumulated higher soluble solids at harvest. This pattern was maintained in 2024 (r = −0.71; R^2^ = 0.51; *p* < 0.001), confirming the stability of the vigor–ripening relationship despite the contrasting climatic conditions between seasons. Similar results were observed in Sauvignon Blanc, where NDVI showed strong negative correlations with °Brix in both years (r = −0.76 in 2023; r = −0.78 in 2024; both *p* < 0.001). These findings reinforce the role of NDVI as a reliable indicator of spatial ripening variability, in line with previous studies demonstrating the sensitivity of NDVI to physiological status and maturity gradients within the vineyard canopy [37,39].

A comparative analysis of all spectral indices also revealed cultivar-specific differences in their predictive performance (Table 1). In both cultivars, NDVI consistently showed the highest explanatory capacity for °Brix, outperforming GNDVI, NDRE, SAVI, MSAVI and OSAVI in every season. In Chardonnay, NDVI displayed the strongest associations with grape maturity in 2023 and 2024, which reflects the stronger canopy density and structural homogeneity of this cultivar. Conversely, Sauvignon Blanc exhibited a more complex spectral behavior: although NDVI remained the best predictor of °Brix, indices with soil-adjustment components (SAVI, OSAVI, MSAVI) displayed comparatively higher associations with other maturity parameters, specifically titratable acidity and pH, in this variety, particularly in 2024 (R^2^ = 0.54–0.57). This response is consistent with the more open canopy architecture of Sauvignon Blanc, which increases soil background visibility and therefore enhances the performance of soil-adjusted vegetation indices for assessing these specific enological parameters. Similar cultivar-dependent spectral sensitivity has been reported by Karakizi et al. [34] and Galidaki et al. [35], who observed that more porous canopy structures amplify the contribution of soil-adjusted spectral components.

These patterns collectively indicate that NDVI captures the structural and physiological canopy attributes most directly linked to sugar accumulation, whereas soil-adjusted indices can provide complementary information for assessing acidity-related maturity parameters in cultivars with lower leaf area index. Additionally, the significant positive relationship between NDVI and vine yield observed in Chardonnay during 2023 suggests that NDVI integrates the major physiological processes connecting vegetative growth, reproductive potential, and fruit maturation. High-vigor vines tend to present greater crop load and delayed sugar accumulation, whereas low-vigor areas achieve faster ripening, a dynamic consistently reflected in the NDVI–Brix relationship. This physiological trade-off aligns with classical vigor–yield–maturation interactions described in precision viticulture literature

A major contribution of this study is the demonstration that the performance of vegetation indices is highly cultivar-dependent. Despite the availability of multiple spectral indices, NDVI consistently emerged as the most robust predictor of grape maturity (°Brix) across both varieties and seasons. The integration of high-resolution UAV imagery with vine-level segmentation enabled the detection of fine-scale vigor–ripening relationships that had not been previously quantified in these cultivars.

However, the comparative analysis revealed a key nuance: while NDVI was the best predictor for sugar accumulation, the soil-adjusted indices (SAVI, OSAVI, MSAVI) showed stronger associations with the spatial patterns of vegetative vigor itself in Sauvignon Blanc (as visually and statistically defined by the k-means clustering of NDVI values). This is evidenced by their higher performance in replicating the predefined vigor zones in this cultivar, particularly in 2024. This enhanced performance of soil-adjusted indices in Sauvignon Blanc is logically explained by its more open canopy structure, which increases soil background visibility and, consequently, the utility of indices designed to minimize this effect. These findings emphasize that while a single index like NDVI may be sufficient for predicting a specific maturity parameter, selecting cultivar-specific spectral indicators is crucial for accurately interpreting overall vegetative status and designing robust ripeness zoning strategies. The demonstrated linkage between canopy vigor and sugar accumulation provides a physiological basis for improving harvest planning and decision-making in precision viticulture [23,40].

The strong and cultivar-specific association between vegetative vigor (as captured by the spatial patterns in NDVI maps) and grape maturity suggests that the underlying physiological status of the vine could also be linked to the biochemical characteristics of must and wine. Since vine stress status, often reflected in lower vigor, is a known driver of pathogenesis-related (PR) protein synthesis, we hypothesized that the spatial patterns of vigor identified spectrally would extend to key oenological attributes. In this context, it became pertinent to examine whether these same vigor zones were associated with wine composition, particularly protein stability and bentonite requirements, which may be similarly influenced by the vegetative status of the vine.

### 2.3. PR-Protein Accumulation and Bentonite Requirement According to Vine Vigor

The accumulation of pathogenesis-related (PR) proteins showed a clear and consistent dependency on vine vigor across cultivars and vintages. Musts obtained from low-vigor vines identified through NDVI-based vigor zoning, systematically exhibited higher concentrations of thaumatin-like proteins (VVTL1–VVTL3) and chitinases (ChiA), whereas musts from high-vigor vines contained substantially lower levels. These biochemical differences were directly reflected in wine instability and bentonite demand (Table 2), indicating that remotely sensed vigor classes are consistently associated with differences in enological stability. Although previous studies have associated PR-protein accumulation with abiotic stressors such as water deficit or elevated temperatures [41,42], none has related these responses to spatial variability in vine vigor within commercial vineyard blocks. Thus, our findings offer the first vineyard-scale evidence linking UAV-derived vigor differences with PR-protein abundance and subsequent fining requirements.

It should be acknowledged that yeast assimilable nitrogen (YAN), a parameter known to influence fermentation kinetics and yeast metabolism [43,44]. was measured during vinification and actively standardized across all treatments. YAN was determined using the o-phthaldialdehyde (NOPA) assay, and diammonium phosphate (DAP) was uniformly added to all fermentations to ensure YAN levels above 200 mg L^−1^, thereby avoiding differential nitrogen availability among vigor zones and cultivars. This controlled nitrogen management minimizes the potential confounding effect of yeast nutrition on fermentation behavior and protein expression.

Consequently, the protein instability patterns observed in this study are interpreted as being primarily associated with in-vineyard physiological processes rather than fermentation-related nitrogen limitations. In addition, no proteolytic enzymes or alternative fining strategies, such as protease-heat or protease-ultrafiltration treatments previously reported to hydrolyze haze-forming proteins post-fermentation, were employed during winemaking [45,46]. This deliberate absence of technological intervention reinforces that the observed differences in protein instability and bentonite requirement reflect spatial variability in vine vigor rather than the effects of targeted oenological practices. Future studies integrating molecular or hormonal indicators of nitrogen and stress regulation would further refine the mechanistic understanding of the interactions between vine vigor, nitrogen status, and PR-protein-mediated instability.

The quantitative patterns of turbidity and bentonite dosage further reinforce the vigor-dependent nature of PR-protein behavior. In Chardonnay 2023, wines from high-vigor vines showed minimal instability (0.69 ± 0.02 ΔNTU), whereas turbidity increased more than 35-fold in the low-vigor sector (25.3 ± 0.7 ΔNTU), resulting in a corresponding rise in bentonite requirement from 200 to 1100 mg L^−1^. Under the milder and more humid conditions of 2024, instability values were markedly lower (1.31–3.46 ΔNTU), highlighting the strong modulation of PR-protein expression by environmental conditions. This seasonal attenuation aligns with earlier observations linking PR-protein synthesis to climatic stress intensity [47,48].

In Sauvignon Blanc, vigor-associated differences were even more pronounced. In 2023, turbidity increased from 8.4 ± 0.3 ΔNTU in high-vigor vines to 54.3 ± 0.3 ΔNTU in low-vigor vines, accompanied by a rise in bentonite requirement from 750 to 1500 mg L^−1^. Although absolute instability values were higher in 2024, the vigor gradient remained significant, reflecting the greater physiological sensitivity of this cultivar to PR-protein accumulation. This cultivar-dependent behavior agrees with reports describing higher baseline concentrations of haze-active proteins in aromatic varieties such as Sauvignon Blanc [12,49].

**Figure 3:**
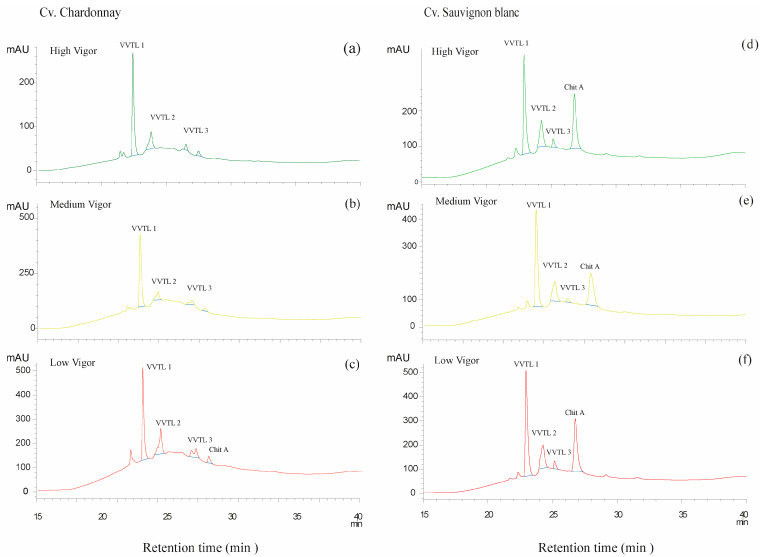
Chromatographic profiles of pathogenesis-related (PR) proteins in free-run juice of *Vitis vinifera* cultivars under three canopy vigor levels. Panels (**a**), (**b**), and (**c**) correspond to Chardonnay samples from high, medium, and low vigor zones, respectively, while panels (**d**), (**e**), and (**f**) show the corresponding profiles for Sauvignon Blanc. PR proteins were separated by reversed-phase HPLC and monitored at 210.4 nm. Major peaks correspond to thaumatin-like proteins (VVTL1–3) and chitinases (ChiA). Higher peak intensities were observed in low-vigor samples (**c**,**f**), particularly in Sauvignon Blanc, indicating increased accumulation of PR proteins under reduced vegetative growth conditions.

The chromatographic signatures obtained by reversed-phase HPLC provide further biochemical confirmation of these vigor-dependent patterns. As illustrated in Figure 3, peak intensities corresponding to VVTL1–3 and ChiA increased systematically from high- to low-vigor zones in both cultivars, fully consistent with the quantitative concentrations summarized in Table 3. The effect was particularly pronounced in Sauvignon Blanc, where low-vigor chromatograms displayed markedly elevated VVTL1 and ChiA peaks, indicative of stronger activation of stress-regulated defense pathways. Chardonnay, in contrast, showed simpler chromatographic patterns and more moderate vigor-related contrasts. These cultivar-specific signatures agree with previous studies reporting distinct PR-protein profiles across white cultivars [48], but our results extend this knowledge by demonstrating that such biochemical differences are not random: they follow a spatially structured gradient defined by vine vigor.

The chromatographic complexity observed in low-vigor vines also provides insight into the qualitative modulation of PR-protein composition. Beyond higher total concentrations, low-vigor chromatograms exhibited broader sets of partially overlapping VVTL and ChiA peaks, suggesting a more heterogeneous mixture of stress-induced isoforms. This pattern was especially evident in Sauvignon Blanc, consistent with its higher sensitivity to abiotic stress. From a physiological standpoint, these differences likely reflect the influence of microclimatic factors, such as increased radiation exposure, higher berry temperature, and reduced water availability, on the induction of PR-protein biosynthesis [45].

Taken together, these results provide the first evidence that spatially explicit canopy vigor measured through NDVI is directly associated with PR-protein accumulation, and consequently, with wine protein instability. The strong agreement between vigor zoning, the chromatographic intensity of specific PR proteins (VVTL1–3 and ChiA), and bentonite requirements indicates that physiological heterogeneity within vineyards can be translated into predictable enological responses. This observation is consistent with the concept of zonal winemaking described by Dorin et al. [18], who demonstrated that vigor-based zoning in Riesling vineyards could differentiate chemical and sensory wine attributes. However, while that study identified enological differences between vigor-defined sectors, it did not elucidate the biochemical mechanisms behind them. In contrast, our results extend current knowledge by providing vineyard-scale evidence that remotely sensed vigor is strongly associated with specific PR-protein families involved in haze formation, as we found significantly higher concentrations of VVTL1, VVTL2, VVTL3, and ChiA in juices from low-vigor zones, which was consistently accompanied by elevated heat-induced turbidity (ΔNTU) and increased bentonite demand in the corresponding wines.

From an enological perspective, the alignment between vigor-dependent PR-protein accumulation and bentonite requirements underscores the practical relevance of vigor zoning. Wines from low-vigor zones consistently required higher fining doses due to their elevated haze-forming potential, a finding that confirms the biochemical patterns observed in this study and is consistent with previous research [2,42]. Importantly, the integration of UAV imagery with biochemical analyses demonstrates that these enological outcomes can be spatially anticipated. Identifying low-vigor sectors early in the season enables wineries to optimize fining protocols, reduce the risks of over-fining with bentonite, and preserve the sensory integrity of white wines. In this sense, vigor-based zoning emerges as a practical, physiologically informed framework that strengthens the links between vineyard variability and winery decision-making within a precision enology context.

### 2.4. Discriminatory Analysis Between Vigor Zones and Enological Parameters

**Figure 4:**
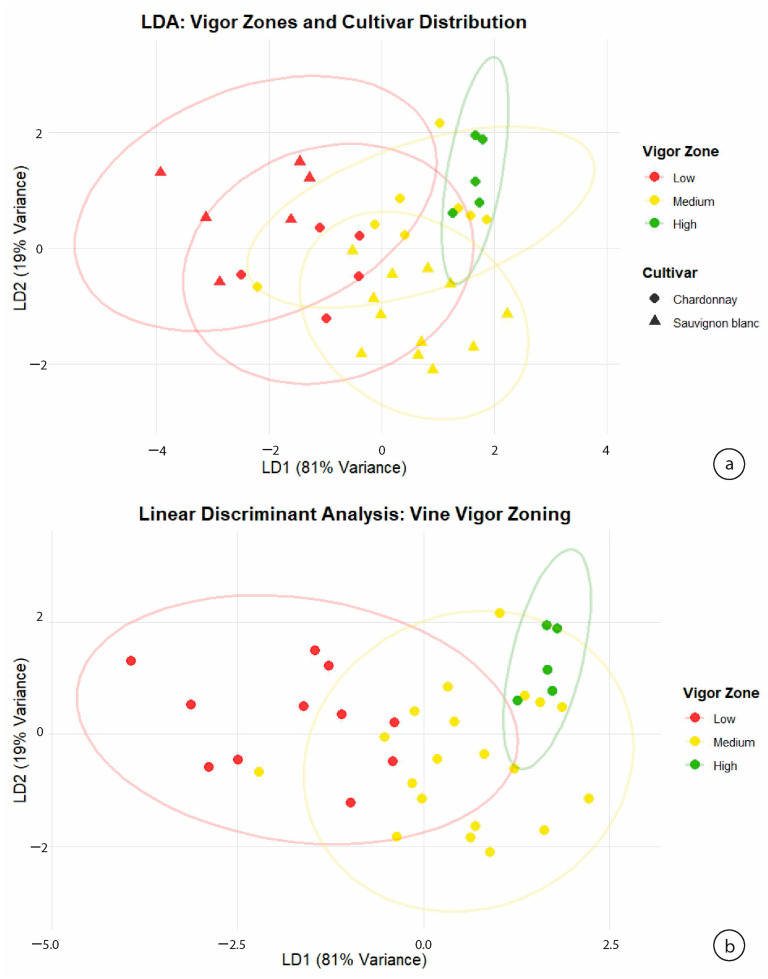
(**a**) Linear Discriminant Analysis (LDA) score plot showing separation of low, medium, and high vigor zones based on PR-protein composition in Chardonnay and Sauvignon Blanc. LD1 and LD2 explain 90% and 10% of variance, respectively. (**b**) Partial Least Squares Discriminant Analysis (PLS-DA) showing similar discriminatory structure, with Component 1 explaining 74.8% and Component 2 explaining 10.3% of variance. Colors indicate vigor zones; shapes indicate cultivars.

Evaluate whether the biochemical composition of PR proteins differed among the vigor zones defined via NDVI, two supervised multivariate methods (Linear Discriminant Analysis, LDA, and Partial Least Squares Discriminant Analysis, PLS-DA) were applied. Both approaches showed strong discriminatory capacity, indicating that PR-protein profiles are associated with physiologically meaningful patterns linked to spatial variability in vine vigor. Figure 4 presents the separation patterns obtained using both multivariate models.

The LDA model developed using the five PR-protein variables (VVTL1, VVTL2, VVTL3, Chitinase A, Chitinase B) explained 90% of the variance in LD1 and 10% in LD2, providing a clear separation among low, medium, and high vigor vines (Figure 4a). LD1 primarily differentiated low-vigor plants, which consistently exhibited elevated concentrations of thaumatin-like proteins and chitinases. This pattern is consistent with previous evidence indicating that PR proteins tend to accumulate under stress conditions such as water deficit, elevated temperature, oxidative stress, or pathogen pressure [1,2]. Low-vigor vines typically experience greater radiation exposure, increased canopy temperatures, and reduced humidity, conditions that have been associated with the activation of stress-related signaling pathways linked to PR-protein synthesis.

The discriminatory performance observed here is consistent with findings by Salazar et al., [46] who demonstrated that PR-protein composition enables accurate classification of grape juices by cultivar, clone, and production zone using LDA. Their work showed that PR proteins provide a strong biochemical fingerprint, and the present study extends this concept by indicating that these proteins can discriminate among physiological environments within the same vineyard, reflecting spatial differences in microclimate and stress.

PLS-DA confirmed the separation observed in LDA. Component 1 explained 74.8% of the variance and separated low-vigor vines from the other classes, while Component 2 (10.3%) distinguished medium- from high-vigor vines (Figure 4b). The clustering pattern suggests an association between reduced canopy density and higher PR-protein levels, whereas high-vigor vines (with cooler, shaded, and more buffered canopies) exhibit lower PR-protein levels.

Cross-validation using a 5-fold M-fold scheme produced classification error rates ranging from 38.9% to 44.4%. These values are expected for physiological classifications in commercial vineyards, where vigor gradients are continuous rather than discrete, leading to partial class overlap and preventing unrealistically high classification accuracy. Importantly, both models achieved meaningful discrimination using only five biochemical predictors, reinforcing the robustness of PR proteins as indicators of vine stress intensity.

All micro-vinification replicates were included in the multivariate analyses to characterize within-zone variability; however, the interpretation focuses on zone-level separation patterns rather than on independent sample classification.

The combined results of LDA and PLS-DA demonstrate that NDVI-based vigor zoning is strongly associated with PR-protein composition at harvest. This relationship is consistent with the biochemical role of PR proteins as markers of abiotic and biotic stress, which is regulated through ABA-, ethylene-, jasmonate-, and salicylic acid-mediated pathways. This indicates that physiological stress patterns captured by NDVI are reflected in differences in PR-protein composition with clear oenological relevance.

These findings also hold significant enological relevance. Low-vigor zones were associated with musts showing higher PR-protein concentrations, increasing the risk of haze formation and requiring greater bentonite additions, in agreement with the observations of [7,42]. Medium-vigor zones showed intermediate biochemical values, while high-vigor zones presented the lowest instability potential. This stratification supports the development of precision stabilization strategies, where oenological interventions such as bentonite fining can be spatially optimized according to local instability risk.

The results obtained here complement prior works where PR-protein variability was attributed to cultivar, climate, or geographic origin. However, this study is the first to demonstrate a clear association between PR-protein discrimination and vigor zoning derived from UAV multispectral data, establishing a physiological and spatial bridge between vineyard variability and protein instability in wine.

## 3. Materials and Methods

### 3.1. Location

**Figure 5:**
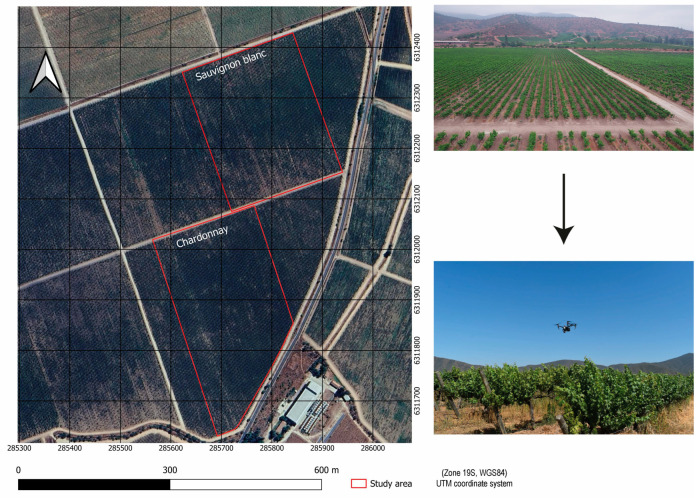
Location of the study area for Sauvignon blanc and Chardonnay vineyards. Coordinates are shown in the UTM coordinate system (meters).

The experiment was conducted during the 2023 and 2024 growing seasons in the Ventisquero vineyard (33°18′40″ S, 71°18′06″ W), located in the Casablanca Valley, Valparaíso Region, Chile (Figure 5). The study site lies at approximately 290 m above sea level and includes two commercial vineyard blocks: one planted with Vitis vinifera cv. Chardonnay (7.2 ha) and the other with V. vinifera cv. Sauvignon Blanc (7.0 ha). Vines were trained on a vertical shoot positioning (VSP) trellis system, spaced 2.50 m between rows and 1.20 m between vines, with rows-oriented east–west. Irrigation was supplied through a drip system using two emitters per vine (3 L h^−1^ each). All agronomic practices, including irrigation scheduling, fertilization, canopy management, and pest and disease control, were entirely managed by the winery according to their standard commercial protocols.

The measurements performed in this study reflected the natural spatial variability of vine vigor within each vineyard block under real commercial conditions, rather than the result of experimental manipulation of the site.

### 3.2. UAV Data Collection

Vine vigor was assessed over two consecutive growing seasons, from December 2022 to March 2023, and from December 2023 to March 2024. A total of ten UAV flights was strategically conducted across the seasons during key phenological stages, ranging from flowering to harvest. All missions were performed using a DJI Inspire 2 unmanned aerial vehicle (DJI Technology Co., Ltd., Shenzhen, China) equipped with a MicaSense RedEdge-MX multispectral camera (MicaSense Inc., Seattle, WA, USA), capturing imagery in five discrete spectral bands: Blue (475 nm), Green (560 nm), Red (668 nm), Red Edge (717 nm), and Near-Infrared (840 nm).

The flights were conducted around local solar noon (12:00–13:00, UTC−4) under clear sky conditions to achieve consistent illumination conditions and minimize the bidirectional reflectance effects. Each mission followed the same flight parameters and acquisition protocols throughout both seasons, which guaranteed temporal comparability and radiometric uniformity.

During image acquisition, the UAV operated at a flight altitude of 75 m above ground level (AGL) and a ground speed of 6 m·s^−1^, with 75% front overlap and 80% side overlap between consecutive images. This configuration provided sufficient coverage for generating high-quality orthomosaics suitable for fine-scale spatial analyses. The RedEdge-MX sensor delivers a ground sampling distance (GSD) of approximately 5 cm·pixel^−1^, enabling precise detection of within-field variability at the vine scale.

Before each flight, we performed radiometric calibration using the standard reflectance panel supplied with the RedEdge-MX system. The onboard Downwelling Light Sensor (DLS2) also continuously recorded ambient light intensity and solar irradiance for all five bands, enabling reliable radiometric normalization during post-processing. For geometric correction, we deployed five ground control points (GCPs) per vineyard plot and georeferenced them precisely using a real-time kinematic (RTK) GNSS station. These GCPs provided sub-decimeter positional accuracy, which established direct spatial correspondence between the UAV imagery and the field-sampled vines.

This standardized methodology for flight timing, sensor calibration, and geometric referencing effectively minimized illumination and terrain-induced artifacts. The resulting consistency enhanced the reliability and comparability of our vegetation indices and vigor zoning across both seasons.

### 3.3. Data Processing and Vigor Classification

We processed the multispectral UAV imagery through a standardized photogrammetric workflow to generate radiometrically consistent and geometrically accurate orthomosaics for the spatial classification of vine vigor. We performed image reconstruction and georeferencing using Agisoft Metashape Professional (version 1.6.3, Agisoft LLC, St. Petersburg, Russia) [50] and conducted the subsequent spectral analysis and vigor classification in QGIS version 3.34 (QGIS Development Team, 2024).

Raw images acquired by the MicaSense RedEdge-MX sensor were radiometrically calibrated using a certified reflectance panel and metadata from the Downwelling Light Sensor (DLS2). This calibration corrected for variable illumination and ensured consistency of reflectance values across acquisition dates. Following the procedure described by Gong et al. [51], band alignment was applied to produce co-registered multispectral composites with sub-pixel precision.

A Structure-from-Motion (SfM) workflow was then implemented to reconstruct the 3D geometry of the vineyard canopy. Five RTK-GNSS-referenced Ground Control Points (GCPs) per block were incorporated during bundle adjustment, yielding sub-decimetric spatial accuracy. The dense point cloud generated from this process was classified into ground and non-ground points, enabling the derivation of a Digital Surface Model (DSM) and a Digital Elevation Model (DEM), both interpolated at 10 cm spatial resolution, following vineyard-proven procedures [28,38].

Subsequently, a Canopy Height Model (CHM) was computed as the difference between the DSM and the DEM, expressed as:

(1) CHM=DEM−DSM

The CHM was used to define a binary vegetation mask isolating pixels corresponding to the vine canopy. This was achieved through a dual-threshold segmentation combining a minimum canopy height of 0.5 m (from CHM) and an NDVI threshold greater than 0.4. This filtering effectively removed non-vegetative elements such as soil, shadows, and trellis structures. Dual-masking methods of this type have been widely validated in UAV-based viticulture studies for improving vegetation segmentation accuracy.

Using the masked multispectral orthomosaic (5 cm pixel^−1^ resolution), a suite of vegetation indices was calculated in QGIS with the Raster Calculator. The indices included NDVI (Normalized Difference Vegetation Index), NDRE (Normalized Difference Red Edge Index), GNDVI (Green NDVI), SAVI (Soil-Adjusted Vegetation Index), OSAVI (Optimized SAVI), MSAVI (Modified SAVI), and TCARI (Transformed Chlorophyll Absorption in Reflectance Index), as summarized in Table 4, which lists their respective equations and references.

Estimation of vine vigor at the individual-plant level was achieved by applying a regular spatial grid (1.20 × 1.00 m) precisely aligned with the vineyard’s planting geometry (2.50 m inter-row and 1.20 m intra-row spacing). Each grid cell corresponded to the canopy footprint of a single vine, from which the mean NDVI value per plant was extracted. This approach minimized intra-row spectral noise and enabled a direct linkage between remotely sensed vigor and enological variables measured at the vine scale.

#### 3.3.1. Selection of Vegetation Index for Zoning

A preliminary exploratory analysis was conducted to evaluate the relationship between the vegetation indices (VIs) derived from UAV multispectral imagery and key enological parameters measured during the two study vintages. Among the calculated indices, the Normalized Difference Vegetation Index (NDVI) consistently exhibited the strongest correlation with grape maturity, particularly with soluble solids (°Brix), showing a moderate-to-strong negative association across both cultivars.

Given its robust relationship with maturity and its widespread validation as a proxy for canopy vigor in viticulture, NDVI was selected as the primary vegetation index for delineating vigor zones. This ensured that the zoning framework reflected physiologically meaningful variability relevant to grape composition and subsequent enological behavior.

#### 3.3.2. Vigor Zoning and Vine Selection

The spatial variability of vine vigor was analyzed by applying a k-means clustering algorithm (k = 3) to NDVI values extracted at the individual vine level. This unsupervised classification segmented each vineyard into three distinct vigor zones (high, medium, and low) based solely on spectral variability patterns.

The choice of k = 3 was guided by both agronomic interpretation and the statistical distribution of NDVI values. The optimal number of clusters was verified through visual inspection of NDVI histograms and the Elbow method, confirming that three groups captured the dominant structure of variability without overfitting. This approach allows for adaptive zoning that reflects the natural heterogeneity of vine vigor rather than imposing arbitrary percentile divisions.

Clustering was conducted independently for each cultivar and growing season to account for interannual differences in canopy structure and phenological development. Although this method produces cluster boundaries that vary slightly between years, it provides a more physiologically meaningful classification of vigor, enabling direct interpretation in relation to vine function and grape composition. This methodology aligns with previous UAV-based viticulture studies that validate NDVI-driven k-means clustering as a robust tool for vineyard zoning and site-specific management [18,57]

**Figure 6:**
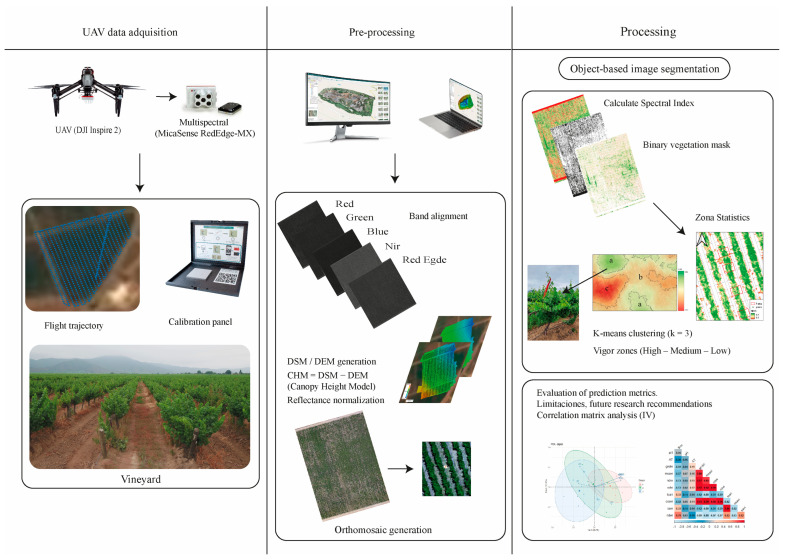
Adapted multispectral image processing workflow and vigor classification [25].

The complete workflow used for UAV-based multispectral data acquisition, pre-processing, and vigor classification is summarized in Figure 6. This diagram integrates the operational steps described above, starting from UAV flight planning and multispectral image acquisition, followed by radiometric and geometric corrections, orthomosaic generation, canopy segmentation through CHM and NDVI thresholds, and the computation of vegetation indices. These successive stages culminate in the k-means–based vigor zoning used for vine selection and subsequent enological analyses. The schematic provides a visual overview of the data pipeline and supports a clearer understanding of the methodological framework applied in this study.

### 3.4. Grape Sampling, Maturity Assessment, and Micro-Vinification

#### 3.4.1. Sampling for Maturity Correlation and Must Preparation

Sampling was conducted one week before the commercial harvest, coinciding with technological maturity (19–21 °Brix). A total of 90 georeferenced vines per cultivar (30 vines from each NDVI-derived vigor zone: high, medium, and low) were harvested. Grapes from the 30 vines within each zone were pooled and homogenized to obtain a representative must sample of approximately 20 kg per vigor zone.

All vines were georeferenced using a real-time kinematic (RTK) GNSS system, ensuring centimeter-level positional accuracy and allowing direct spatial linkage between each vine and its vigor classification derived from UAV multispectral imagery.

At harvest, grape composition and yield parameters were evaluated on the same vines. The measurements included total soluble solids (°Brix) using a digital refractometer, titratable acidity (TA, g L^−1^ as tartaric acid) by titration, and pH using a calibrated pH meter. In addition, mean bunch weight (g cluster^−1^) and yield per vine (kg vine^−1^) were calculated from the number of clusters per plant and their average weight. These data were averaged per vigor zone (n = 30 vines) to evaluate the effect of vine vigor on grape maturity and productivity at harvest.

#### 3.4.2. Micro-Vinification Protocol

At the pilot winery of the Pontificia Universidad Católica de Valparaíso, grape clusters were destemmed using an electric destemmer and pressed to obtain free-run juice, which was filtered through a 0.45 µm membrane to remove suspended solids. Potassium metabisulfite (75 mg L^−1^) was added as an antioxidant and antimicrobial agent prior to fermentation.

For each cultivar, year, and vigor zone, grapes from 30 vines were harvested and pooled to generate a single composite must per zone. This pool must constitute the biological experimental unit. Three independent micro-vinifications were then conducted from each pooled must to evaluate technical and fermentation-related variability. These micro-vinifications were therefore considered technical replicates and not independent biological replicates.

The clarified must was transferred into 2 L glass fermentation bottles, with three technical replicates per vigor class, yielding a total of nine fermentations per cultivar (n = 9) corresponding to the NDVI-defined vigor levels (high, medium, and low). This procedure was applied separately to both Chardonnay and Sauvignon Blanc in each vintage. Fermentation was initiated by inoculating the must with *Saccharomyces cerevisiae* ZYMAFLORE^®^ VL3 (Laffort), a strain selected for white-wine production due to its ability to enhance thiol-type varietal aromas and maintain balanced fermentation kinetics. The yeast was rehydrated according to the manufacturer’s instructions and inoculated at 20–30 g hL^−1^. All fermentations were conducted at 15 °C, within the optimal temperature range (15–21 °C) for this strain.

Yeast assimilable nitrogen (YAN) was determined during vinification using the o-phthaldialdehyde (NOPA) assay, following the methodology described by Salazar et al. [58]. Based on these measurements, diammonium phosphate (DAP) was added on the third day of fermentation at a dose of 20 g hL^−1^ to standardize nitrogen availability and ensure YAN levels above 200 mg L^−1^, considered adequate for complete and balanced alcoholic fermentation. This nitrogen adjustment was applied uniformly across all cultivars, vintages, and vigor zones to avoid confounding effects related to differential nitrogen availability.

Fermentation progress was monitored daily by measuring must density with an electronic densimeter, and completion was confirmed when density remained stable at approximately 0.995 g mL^−1^ for three consecutive days. The resulting wines were cold-stabilized at −2 °C for 10 days to promote tartaric and protein precipitation, followed by bentonite fining and sterile filtration through 0.45 µm cellulose membranes. Bottled wines were labeled by variety, vintage, and vigor class and stored at 10–12 °C until subsequent chemical and protein analyses.

### 3.5. Chemical Composition and Protein Stability Analysis

#### 3.5.1. pH and Titratable Acidity

The pH was determined using a digital pH meter, calibrated with standard buffer solutions at pH 4 and 7 Titratable acidity (TA) was expressed as grams of tartaric acid per liter and determined by titration with 0.1 N NaOH using phenolphthalein as the endpoint indicator. A known volume of wine was titrated until a persistent pale pink color was observed, indicating pH~8.2.

#### 3.5.2. Polyphenol Content (GAE)

The total phenolic content was determined using a microplate-adapted Folin-Ciocalteu colorimetric assay [59], with modifications from previous studies Lee et al., (2014) [60]. Briefly, 10 μL of the wine sample was mixed with 75 μL of distilled water and 20 μL of 2N Folin-Ciocalteu reagent in a 96-well microplate. After a 5-min incubation in darkness, 30 μL of 10% sodium carbonate (Na_2_CO_3_) and 120 μL of distilled water were added. The plate was then incubated for 1 h at room temperature in darkness prior to absorbance measurement.

Absorbance was measured at 760 nm using a UV–visible microplate reader. A calibration curve was constructed using gallic acid at concentrations ranging from 0 to 1000 µg/mL, prepared by dissolving 12.5 mg of gallic acid in 5 mL of methanol and performing serial dilutions. Final results were expressed as gallic acid equivalents (GAE) in µg/mL [61].

#### 3.5.3. PR-Protein Quantification by Reversed-Phase HPLC

The composition of pathogenesis-related (PR) proteins in grape juice was determined by reversed-phase high-performance liquid chromatography (RP-HPLC), according to Salazar et al. [62] After filtration through 0.45 mm pore size membranes, 20 mL aliquots of grape juice sample was loaded at 0.2 mL min^−1^ onto a Waters XBridge TM BEH300 C4 RP column (2.1 × 150 mm; 3.5 µm particle size), equilibrated in a mixture composed of 17% (*v*/*v*) solvent A 80% (*v*/*v*) acetonitrile and 0.1% (*v*/*v*) trifluoroacetic acid] and 83% solvent B [8% (*v*/*v*) acetonitrile and 0.1% (*v*/*v*) trifluoroacetic acid] and held at 40 °C. Proteins were then eluted using a multi-step gradient of solvent A from 17% solvent A to 49% solvent A in the first 4.5 min, from 49 to 57% between 4.5 and 7.5 min, from 57 to 65% between 7.5 and 8.5 min, from 65 to 81% between 8.5 and 22.5 min, held at 81% until 27.5 min, from 81 to 17% between 27.5 and 33.5 min, and held at 17% until 35.5 min. Proteins were detected at 210.4 and 220.4 nm by the DAD detector. Protein identities were assigned by comparison of their retention times with those previously reported by different laboratories [1,7,12,42] and by comparison against a commercial thaumatin-like protein standard (Sigma-Aldrich, St Louis, MO, USA).

#### 3.5.4. Protein Instability by Heat Test

Protein instability was evaluated using a heat stability test, following the methodology described by Salazar et al. [62] for the assessment of protein haze formation in Sauvignon Blanc and Chardonnay wines from the Casablanca Valley. This heat test is one of the most widely adopted approaches in both enological research and winery practice for evaluating protein instability in white wines, as summarized in recent reviews on wine protein haze formation. This test simulates the thermal denaturation and aggregation of thermolabile pathogenesis-related (PR) proteins, which are the main contributors to protein haze formation in bottled white wines.

For each analysis, 20 mL of wine was filtered through a 0.45 µm cellulose acetate membrane to remove suspended solids and minimize background turbidity. The clarified samples were transferred to sealed borosilicate glass tubes and incubated in a thermostatically controlled water bath at 80 °C for 2 h, a temperature–time combination frequently reported as effective for predicting protein instability under accelerated conditions [63].

Immediately after heating, the tubes were rapidly cooled in an ice bath and then allowed to equilibrate to room temperature. Turbidity was measured before and after heat treatment using a calibrated nephelometric turbidimeter (Hanna Instruments, model HI 83749), and results were expressed in nephelometric turbidity units (NTU). Protein instability was calculated as the difference between heated and unheated samples (ΔNTU).

Wines were classified as protein-unstable when ΔNTU exceeded 2.0 NTU, a threshold commonly applied in both experimental studies and commercial wineries to define acceptable protein stability [45,46,48].

#### 3.5.5. Determination of Bentonite Requirement for Protein Stabilization

Determine the minimum dose of bentonite required to stabilize each wine, clarification tests were performed on unclarified samples after the initial thermal stability assessment. Based on the ΔNTU values obtained in the thermal test performed with untreated wines, sodium bentonite was applied in increasing concentrations (50, 100, 200, 300, and 400 mg L^−1^).

The bentonite was pre-hydrated (5% *w*/*v*) in distilled water for 24 h and then thoroughly mixed with 20 mL aliquots of wine. The treated samples were stored at 4 °C for 24 h to allow interaction between the proteins and bentonite and sedimentation. After decanting and filtration, the thermal stability test was repeated under the same conditions described above. The lowest concentration of bentonite that reduced ΔNTU to ≤2.0 was recorded as the minimum effective dose of bentonite, in accordance with standard oenological protocols [42].

#### 3.5.6. Statistical Analyses

Statistical analyses were conducted to assess the influence of vine vigor on protein instability (ΔNTU), individual PR-protein fractions, total PR-protein concentration, and bentonite requirement. One-way analysis of variance (ANOVA) was applied to test for differences among vigor zones (high, medium, and low).

For fermentation-derived variables, the experimental unit corresponded to the pooled must per vigor zone, while the three micro-vinifications conducted per zone were considered technical replicates reflecting fermentation-related variability. Accordingly, inferential statistics were interpreted conservatively, and results are presented as zone-level comparisons rather than independent biological replicates.

Prior to ANOVA, data were evaluated for compliance with the assumptions of normality and homogeneity of variance using the Shapiro–Wilk and Levene tests, respectively. When both assumptions were satisfied, mean separations were performed using Tukey’s HSD post hoc test at *p* < 0.05.

Pearson correlation coefficients were calculated to quantify the relationships between NDVI values, protein instability (ΔNTU), PR-protein accumulation, and bentonite dosage. These correlations were computed at the zone level and interpreted as associative relationships rather than causal effects.

Multivariate analyses were conducted using Linear Discriminant Analysis (LDA) and Partial Least Squares Discriminant Analysis (PLS-DA) based on NDVI, ΔNTU, PR-protein variables, and bentonite requirement. These multivariate approaches were applied in an exploratory framework to visualize patterns of separation among vigor classes and to identify dominant spectral–biochemical associations, rather than to derive definitive classification rules. Cross-validation was performed using a 5-fold scheme to estimate classification error and reduce overfitting.

All statistical procedures were performed using R software version 4.3.1 (R Foundation for Statistical Computing, Vienna, Austria).

## 4. Conclusions

This study demonstrates that UAV-based NDVI vigor zoning is significantly associated with spatial variability in PR-protein accumulation, protein instability, and bentonite requirements in white wines. NDVI-derived vigor classes consistently identified vineyard zones with contrasting levels of haze-forming proteins, highlighting their potential value as early indicators of relative protein instability risk.

Rather than implying a direct causal mechanism, the observed relationships indicate that the NDVI captures underlying physiological and microclimatic conditions associated with vine stress status, which are reflected in downstream enological outcomes. The consistent associations between the NDVI, PR-protein concentration, ΔNTU values, and bentonite demand suggest that UAV-derived vigor maps can be used to predict relative instability risk across vineyard blocks.

From an applied perspective, these findings support the integration of UAV-based vigor mapping into precision-enology workflows, enabling site-specific stabilization strategies such as differential bentonite fining or targeted cellar interventions. By identifying high-risk zones prior to harvest, this approach offers a practical framework to optimize protein stabilization while minimizing excessive fining, wine losses, and potential quality degradation.

Overall, this work provides exploratory but robust evidence that spatial vineyard information can be leveraged to anticipate enological challenges associated with protein instability, reinforcing the role of precision viticulture as a decision-support tool rather than as a deterministic control of wine composition.

## Figures and Tables

**Table 1:** Correlation coefficients (r) and coefficients of determination (R^2^) between UAV-derived spectral indices and grape maturity (°Brix) at harvest for Chardonnay and Sauvignon Blanc during the 2023 and 2024 seasons.

	Chardonnay				Sauvignon Blanc			
Índex	2023		2024		2023		2024	
	r	R^2^	r	R^2^	r	R^2^	r	R^2^
NDVI	−0.85	0.73	−0.71	0.51	−0.76	0.57	−0.78	0.61
GNDVI	−0.77	0.59	−0.69	0.47	−0.61	0.37	−0.61	0.38
NDRE	−0.70	0.49	−0.71	0.51	−0.47	0.23	−0.62	0.39
SAVI	−0.31	0.09	0.32	0.1	−0.75	0.57	−0.43	0.18
OSAVI	−0.50	0.24	−0.61	0.38	−0.75	0.57	−0.52	0.27
MSAVI	−0.29	0.08	−0.56	0.31	−0.73	0.54	−0.43	0.18

Values represent Pearson correlation coefficients (r) and coefficients of determination (R^2^) obtained from simple linear regression models of the form °Brix ~ Index. Negative r values indicate an inverse association between canopy vigor and soluble solids at harvest, meaning higher ripeness in lower-vigor vines. Pearson’s method was selected due to the continuous and approximately linear relationships between spectral indices and °Brix. Coefficients of determination (R^2^) reflect the proportion of variation in °Brix explained by each vegetation index. All variables were evaluated at the vine scale using UAV-based multispectral data segmented at individual-plant resolution.

**Table 2:** Protein instability (ΔNTU) and bentonite dosage (mg/L) required to stabilize Chardonnay and Sauvignon Blanc wines produced from different vigor zones (A: high, B: medium, C: low) during the 2023 and 2024 vintages.

		Protein Instability (ΔNTU)		Bentonite Dosage (mg/L)	
		2023	2024	2023	2024
Chardonnay	Vigor Zone				
	Zone A	0.69 ± 0.02 ^a^	1.31 ± 0.17 ^a^	200 ^a^	150 ^a^
	Zone B	18.24 ± 0.25 ^b^	1.41 ± 0.15 ^a^	750 ^b^	150 ^a^
	Zone C	25.32 ± 0.69 ^c^	3.46 ± 0.13 ^b^	1100 ^c^	300 ^b^
Sauvignon Blanc					
	Zona A	8.43 ± 0.34 ^a^	48.80 ± 0.59 ^a^	750 ^a^	1100 ^a^
	Zone B	29.84 ± 1.43 ^b^	56.12 ± 0.24 ^b^	1100 ^b^	1100 ^a^
	Zone C	54.30 ± 0.33 ^c^	72.58 ± 0.70 ^c^	1500 ^c^	1500 ^b^

Data are expressed as mean ± standard deviation. Different lowercase superscript letters (a, b, c) within each column indicate statistically significant differences (*p* < 0.05) among vigor zones within the same variety and vintage according to Tukey’s HSD test. Higher protein instability and bentonite demand were consistently observed in wines from low-vigor zones, particularly in Sauvignon Blanc.

**Table 3:** Total pathogenesis-related (PR) protein content (VVTL1 + VVTL2 + VVTL3 + ChitA + ChitB, mg L^−1^) according to vine vigor zones for Chardonnay and Sauvignon Blanc wines (2023–2024).

Variety	Vigor Zone	Total, PR (mg L^−1^)	
		2023	2024
	Vigor zone		
Chardonnay	High	37.37 ± 0.68 ^a^	25.97 ± 1.06 ^a^
	Medium	58.90 ± 1.13 ^b^	36.03 ± 1.36 ^b^
	Low	73.87 ± 4.64 ^c^	47.03 ± 3.86 ^c^
Sauvignon Blanc	High	49.47 ± 5.56 ^a^	55.00 ± 1.49 ^a^
	Medium	76.93 ± 2.90 ^b^	83.44 ± 1.98 ^b^
	Low	96.50 ± 3.01 ^c^	108.40 ± 4.45 ^c^

Data represent mean ± standard deviation. Values correspond to the sum of thaumatin-like proteins (VVTL1–3) and chitinases (ChitA and ChitB). Wines from low-vigor zones consistently showed higher PR-protein accumulation, instability, and bentonite demand, particularly in Sauvignon Blanc. Different superscript letters within each variety and vintage indicate statistically significant differences (*p* < 0.05) among vigor zones according to Tukey’s HSD test.

**Table 4:** Spectral vegetation indices used to assess vine canopy vigor and physiological status.

Spectral Index	Formula	Reference
NDVI	((NIR − RED))/((NIR + RED))	[52]
NDRE	(NIR − RedEdge)/(NIR + RedEdge)	[53]
GNDVI	(NIR − Green)/(NIR + Green)	[31]
SAVI	((NIR−Red)/(NIR + Red + L)) × (1 + L)	[54]
MSAVI	$\frac{\left[2\times NIR+1-\sqrt{\left((2\times NIR+1{)}^{2}-8\times \left(NIR-RED\right)\right)}\right]}{2}$	[55]
OSAVI	(NIR − Red)/(NIR + Red + 0.16)	[56]

## Data Availability

The data supporting the findings of this study are available from the corresponding author upon reasonable request. These datasets are currently being used in additional ongoing studies and cannot be shared publicly until those analyses are completed.

## Abbreviations

The following abbreviations are used in this manuscript:

ABA	Abscisic acid
CHM	Canopy height model
CWSI	Crop water stress index
DEM	Digital elevation model
DSM	Digital surface model
GNDVI	Green Normalized Difference Vegetation Index
LAI	Leaf area index
LDA	Linear Discriminant Analysis
NDRE	Normalized Difference Red Edge Index
NDVI	Normalized Difference Vegetation Index
PLS-DA	Partial Least Squares Discriminant Analysis
PR proteins	Pathogenesis-related proteins
SAVI	Soil-Adjusted Vegetation Index
UAV	Unmanned aerial vehicle
